# Sex difference in nocturnal blood pressure dipping in adolescents with varying degrees of adiposity

**DOI:** 10.1186/s12887-024-04804-0

**Published:** 2024-05-22

**Authors:** Yi Zhou, Lin Zhao, Zenglei Zhang, Xu Meng, Qiu-jing Cai, Xiao-lei Zhao, Lin-ping Wang, Ai-hua Hu, Xian-liang Zhou

**Affiliations:** 1 Department of Cardiology, Shenzhen Cardiovascular Minimally Invasive Medical Engineering Technology Research and Development Center, Shenzhen People’s Hospital (The Second Clinical Medical College, Jinan University; The First Affiliated Hospital, Southern University of Science and Technology), Shenzhen, Guangdong 518020 China; 2 Department of Cardiology, Fuwai Hospital, National Center for Cardiovascular Diseases, Chinese Academy of Medical Sciences and Peking Union Medical College, Beijing, 100037 China; 3 National Integrated Traditional and Western Medicine Center for Cardiovascular Disease, China-Japan Friendship Hospital, Beijing, China; 4 Department of Non-communicable Disease Management, Beijing Children’s Hospital, National Center for Children’s Health, Capital Medical University, Beijing, China

**Keywords:** Ambulatory blood pressure monitor, Adolescents, Dipping patterns, Obesity severity, Sex difference

## Abstract

**Background:**

For adolescents, abnormal dipping patterns in blood pressure (BP) are associated with early-onset organ damage and a higher risk of cardiovascular disorders in adulthood. Obesity is one of the most common reasons for abnormal BP dipping in young people. However, it is unknown whether the severity of obesity is associated with BP dipping status and whether this association is sex-dependent.

**Methods:**

499 participants between 12 and 17 years old with overweight or obesity underwent ambulatory blood pressure monitoring (ABPM) between April 2018 and January 2019 in Beijing and Baoding. Participants were grouped by body mass index (BMI) into overweight (BMI 85th–95th percentile), obese (BMI ≥ 95th percentile) and severely obese (BMI ≥ 120% of 95th percentile or ≥ 35 kg/m^2^) groups. Non-dipping was defined as a < 10% reduction in BP from day to night. The interaction effect between sex and obesity degree was also analyzed.

**Results:**

326 boys and 173 girls were included, of whom 130 were overweight, 189 were obese, and 180 were severely obese. Girls with severe obesity had a higher prevalence of non-dipping, but boys showed no significant differences in BP dipping status between obesity categories. In addition, as obesity severity went up, a more evident increase in night-time SBP was observed in girls than in boys.

**Conclusions:**

Severely obese is associated with a higher prevalence of non-BP dipping patterns in girls than in boys, which suggests that the relationship between the severity of obesity and BP dipping status might be sex-specific.

## Introduction

There has been important progress in the field of pediatric hypertension during recent decades, one of which is the growing use of 24-hour ambulatory blood pressure monitoring (ABPM) [1, 2]. By measuring blood pressure (BP) both during the day and night, ABPM permits physicians to evaluate circadian patterns in BP, which should include a natural dip during sleep [3, 4]. A loss or reduction in the nocturnal decline in BP provides significant prognostic information, regardless of the absolute BP value [5]. In adults, abnormal BP dipping patterns could lead to higher risks of heart failure, renal hypofunction and cardiovascular mortality [6–8]. In children and adolescents, non-dippers are also associated with target organ damage (TOD) including left ventricular hypertrophy (LVH), proteinuria, and greater carotid intima-media thickness [9–12]. On the basis of these evidence, current guidelines suggest that abnormal BP dipping status should be given the same weight as abnormalities in BP values when treating pediatric hypertension [12]. 

Multiple risk factors have been shown to be associated with non-dipping patterns and obesity is one of the leading factors in both adults and children [13, 14]. In adults, there is a high prevalence of non-dippers in patients with obesity, and this figure could be higher in those with more severe obesity degree, suggesting that the severity of obesity also affects dipping status [15, 16]. However, it remains unclear whether more severe obesity in young people is associated with a higher risk of non-dipping patterns. A few previous studies on this topic have generated conflicting results due to either small sample sizes or participants with a wide age range, from kindergarten kids to college students [13, 17–19]. In addition, none of the above studies have considered sex differences. Owing to biological and behavioral differences, sex-specific manifestations have been frequently reported in the causes and consequences of obesity in children and adolescents, which implies that boys and girls may face different obesity-related cardiometabolic risks and thus require sex-specific management [20–22]. However, to the best of our knowledge, such potential differences have not been discussed in circadian abnormalities of blood pressure related with adiposity.

To further investigate the relationship between obesity severity and non-BP dipping patterns in young people and to determine whether this association is sex-specific, we performed a cross-sectional study among adolescents with overweight or obesity. Based on current evidence, we hypothesized that more severe obesity is associated with a higher risk of non-BP dipping and this relationship is likely to be gender-dependent.

## Patients and methods

### Study design and participants

The present study was carried out in Beijing and Baoding. Overweight and obese adolescents between 12 and 17 years who were referred to wear an ABPM device produced by Kang Electronics Technology Company between April 2018 and January 2019 were included. The exclusion criteria were as follows: (1) adolescents within the normal range of BMI, which was below the 85th percentile for age and sex [22]; (2) with a known history of the use of antihypertensive drugs or other medication that could affect BP ; (3) with a known history of diseases that could affect BP, including chronic kidney disease, congenital heart disease, and thyroid disorders. The study was conducted in accordance with the principles of the Declaration of Helsinki and was approved by the Ethics Committee of the Beijing Hypertensive League. Informed consent was not required due to the retrospective design and the anonymity of the study data.

### Measurements and definition of obesity severity

Basic demographic information, including age, sex, height, and body mass, was extracted from ABPM reports. Body mass index (BMI) was calculated as body mass (kg) divided by height squared (m^2^). According to the 2017 Endocrine Society Practice Guidelines, we defined adolescents as overweight if their BMI was ≥ 85th percentile but < 95th percentile, as obese if their BMI was ≥ 95th percentile and as severely obese if their BMI was ≥ 120% of the 95th percentile or ≥ 35 kg/m^2^ [23]. The BMI percentile reference values used in the present study were those defined for Chinese adolescents [24]. 

### Measurement and definition of BP

Office blood pressure (OBP) was measured in a sitting position using the right arm by trained medical professionals using an automatic electronic oscillometric sphygmomanometer (Omron HBP-1300, Kyoto, Japan). BP was measured three times at 5-minute intervals and the mean value was recorded. Ambulatory BP was monitored by a portable, non-invasive, automated monitoring and recording system (KC-2300 A; Beijing Kangkang Shengshi Information Technology Co, Ltd, China), which was programmed to record systolic blood pressure (SBP), diastolic blood pressure (DBP), and heart rate (HR) at 30-minute intervals during the daytime (06:00–22:00) and at 60-minute intervals during the night (22:00–06:00). Following the clinical practice guidelines, the non-dominant arm was used during ABPM [1]. Qualified ABPM reports required at least 70% successful readings during the monitoring period and a minimum of one reading per hour.

According to the updated statements published by the American Heart Association (AHA) in 2022 [12], for adolescents ≥ 13 years, normal OBP was defined as < 130/80 mmHg and normal ABP as < 125/75 mmHg over 24 h, < 130/80 mmHg during the daytime, and 110/65 mmHg during the night. For children < 13 years, the 95th percentile for age and sex was used as the diagnostic criterion for both office and ambulatory hypertension.

The circadian pattern of BP was classified according to the percentage decline in BP from daytime to nighttime. To keep in line with the guideline published by European Society of Hypertension (ESH) and previous studies of pediatric hypertension, non-dippers were defined as less than 10% reduction in nocturnal SBP or DBP [14, 17–19, 25]. Isolated nocturnal hypertension (INH) was defined as hypertension during the night with normal daytime BP.

### Statistical analysis

For all characteristics, continuous variables with a normal distribution were expressed as mean ± standard deviation, otherwise were described using median (interquartile range). ANOVA test was used for inter-group comparison for the former and non-parametric tests were applied to the latter. Categorical variables were represented as number (%) and analyzed with Chi-square test or Fisher’s exact test. All statistical analyses were performed using SPSS, Version 26.0 (SPSS, Inc, Chicago, IL), and a two-sided *P* < 0.05 was considered statistically significant.

## Results

**Fig. 1:**
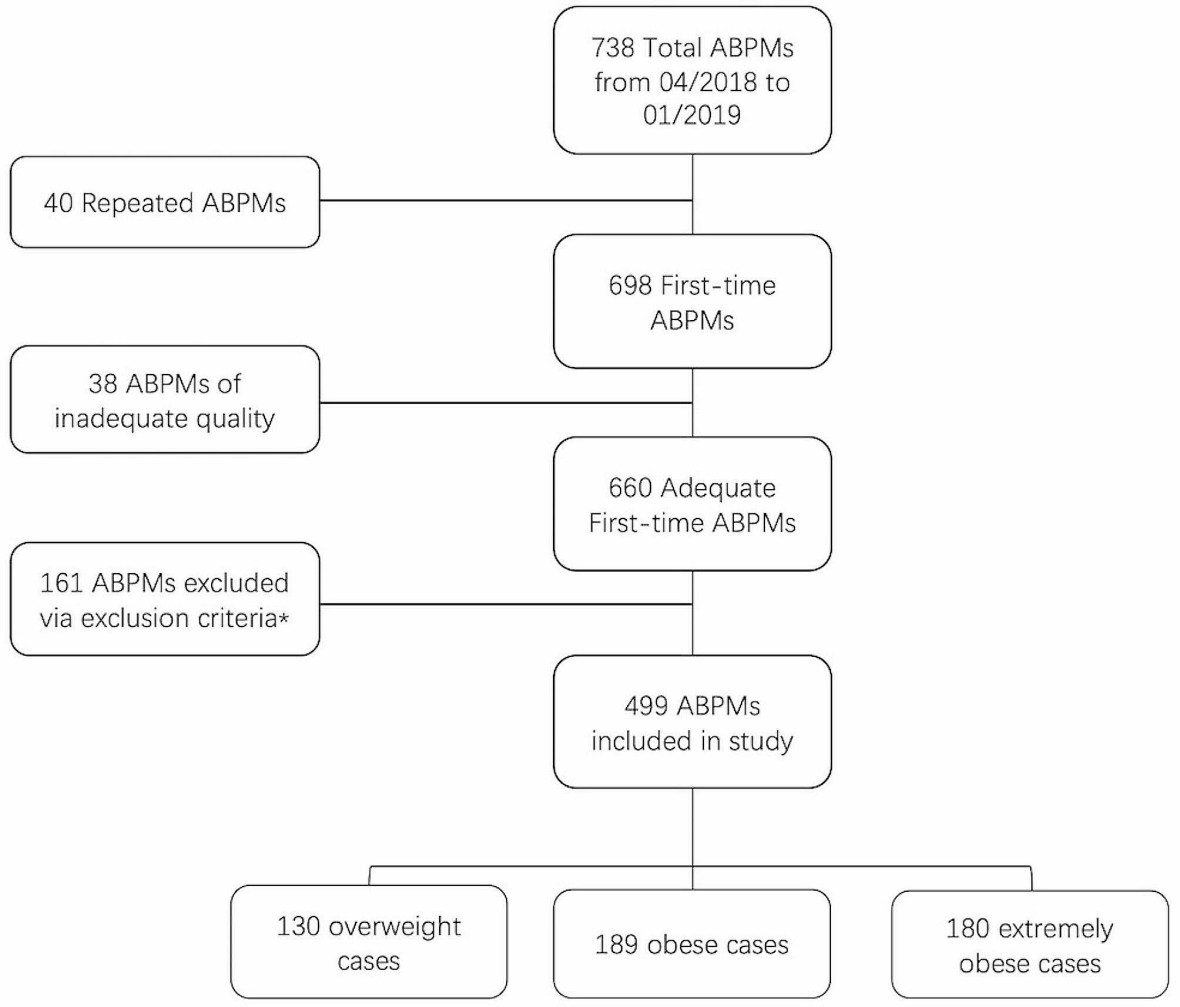
Flow chart of the present study. *160 participants had normal BMI and one was in antihypertensive medication. Abbreviations: ABPM, ambulatory blood pressure monitor. BMI, body mass index

A total of 738 ABPM reports were obtained between April 2018 and January 2019 (Fig. 1). Of these, 40 ABPM reports were repeatedly performed on the same subject, thus 698 “first-time” reports were identified. 38 reports were classified as inadequate quality for interpretation. Among the remaining 660 participants, 160 of them were within a normal range of BMI and one was under antihypertensive medication. Finally, there were 499 adolescents included, of whom 130 were overweight, 189 were obese and 180 were severely obese.

The mean age of all the participants was 14.6 ± 1.6 years and 65.3% of them were boys. The distributions of age and obesity degrees between boys and girls were similar (Table 1). Compared with boys, girls had similar office SBP, but slightly higher office DBP. However, the 24-hour, daytime and nighttime ambulatory SBP were all higher in boys than girls.

**Table 1:** Demographic features and BP parameters of participants grouped by sex

	Overall(*n* = 499)	Boys(*n* = 326)	Girls (*n* = 173)	*P*-value
Age, years	14.6 ± 1.6	14.7 ± 1.6	14.5 ± 1.6	0.190
BMI, kg/m^2^	27.7(24.7, 31.0)	28.3(24.8, 31.1)	26.7(24.5, 29.9)	0.006
Obesity degree,%				0.257
Overweight	130(26.1)	89(27.3)	41(23.7)	
Obese	189(37.9)	115(35.3)	74(42.8)	
Severely Obese	180(36.1)	122(37.4)	58(33.5)	
Office SBP, mmHg	143.6 ± 13.8	144.3 ± 14.1	142.3 ± 11.7	0.114
Office DBP, mmHg	88.1 ± 11.6	87.2 ± 11.7	89.6 ± 11.3	0.026
24 h SBP, mmHg	128.8 ± 9.9	130.5 ± 9.5	125.7 ± 9.8	<0.001
24 h DBP, mmHg	75.0 ± 7.7	75.2 ± 7.7	74.7 ± 7.7	0.531
Day-time SBP, mmHg	131.1 ± 10.0	132.6 ± 9.8	128.2 ± 9.9	<0.001
Day-time DBP, mmHg	77.4 ± 7.9	77.5 ± 7.9	77.3 ± 8.0	0.859
Night-time SBP, mmHg	119.8 ± 11.9	121.6 ± 11.7	116.4 ± 11.5	<0.001
Night-timeDBP, mmHg	65.4 ± 9.2	65.7 ± 9.5	65.0 ± 8.6	0.417
BP Phenotype, %				<0.001
Normotensive	10(2.0)	7(2.1)	3(1.7)	
WCH	55(11.0)	20(6.1)	35(20.2)	
MHT	18(3.6)	16(4.9)	2(1.2)	
SHT	416(83.4)	283(86.8)	133(76.9)	

WCH: white coat hypertension; MHT: masked hypertension; SHT: sustained hypertension

**Fig. 2:**
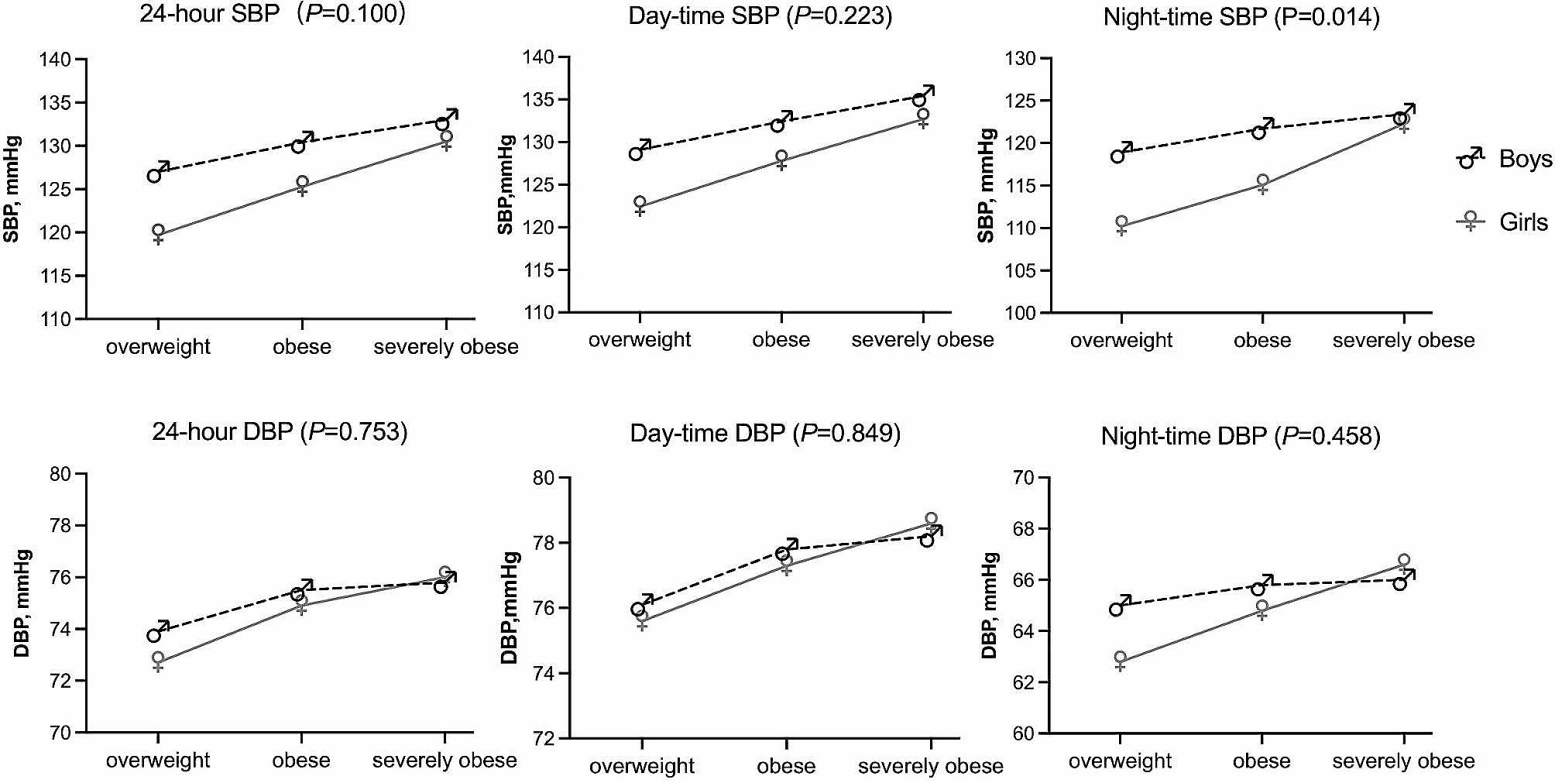
24-hour, day-time and night-time BP in different obesity degree grouped by sex. Two-way ANOVA was conducted to investigate the relationship between obesity degrees and sex on ambulatory BP value, which showed a statistical interaction effect between obesity degree and sex on night-time SBP (*P* = 0.014)

Table 2 shows the ABPM findings in participants of different genders and varying degrees of obesity. Overall, ambulatory SBP elevated with increasing severity of adiposity, while DBP showed no significant difference. As a result, day-night hypertension became more frequent as obesity level increased. However, the prevalence of INH, seemed to decreased as obesity severity went up and such tendency was found in boys(36.0% vs. 28.7% vs. 20.5%, *P* = 0.044) rather than in girls( 22.0% vs. 20.3% vs. 24.1%, *P* = 0.868), which revealed a potential sex difference in the relationship between adiposity level and ambulatory blood pressure. To further investigate whether there was sex-dependent effect, we performed a two-way ANOVA and found a significant interaction between the severity of obesity and sex with respect to nighttime SBP (*P* = 0.014). These results are presented in Fig. 2. As the severity of obesity increased, a more evident rise in nighttime SBP occurred in girls than in boys. However, no significant interactions were observed in other BP parameters.

**Table 2:** ABPM findings in overweight, obese and extreme obese participants stratified by sex

	Overall(*n* = 499)				Boys(*n* = 326)				Girls(*n* = 173)			
	Overweight (*n* = 130)	Obese (*n* = 189)	Se Obese (*n* = 180)	*P*	Overweight (*n* = 89)	Obese (*n* = 115)	Se Obese (*n* = 122)	*P*	Overweight (*n* = 41)	Obese (*n* = 74)	Se Obese (*n* = 58)	*P*
24 h SBP, mmHg	124.7 ± 9.0^+*^	128.4 ± 9.8^^*^	132.2 ± 9.4^^+^	<0.001	127.0 ± 7.8^+*^	130.4 ± 10.1^^^	133.0 ± 9.3^+^	<0.001	119.7 ± 9.6^*+^	125.3 ± 8.4^*^^	133.5 ± 9.4^+^^	<0.001
24 h DBP, mmHg	73.5 ± 7.6^*^	75.3 ± 8.0	75.9 ± 7.3^^^	0.028	73.9 ± 7.1	75.5 ± 8.3	75.8 ± 7.5	0.153	72.7 ± 8.5	74.9 ± 7.6	76.0 ± 6.9	0.113
Day-time SBP, mmHg	127.0 ± 9.2^+*^	130.6 ± 9.8^^*^	134.5 ± 9.6^^+^	<0.001	129.1 ± 8.2^+*^	132.4 ± 10.2^^*^	135.4 ± 9.6^^+^	<0.001	122.4 ± 9.7^*+^	127.8 ± 8.4^*^^	132.7 ± 9.6^+^^	<0.001
Day-time DBP, mmHg	76.0 ± 7.9^*^	77.6 ± 8.3	78.3 ± 7.5^^^	0.035	76.1 ± 7.6	77.8 ± 8.4	78.2 ± 7.6	0.141	75.6 ± 8.5	77.3 ± 8.2	78.6 ± 7.3	0.191
Night-time SBP, mmHg	116.1 ± 11.0^*^	119.2 ± 12.1^*^	123.0 ± 11.5^^+^	<0.001	118.9 ± 9.9^*^	121.7 ± 12.5	123.4 ± 11.9^^^	0.011	110.2 ± 11.1^*^	115.1 ± 10.4	122.3 ± 10.5^^^	<0.001
Night-time DBP, mmHg	64.3 ± 8.9	65.4 ± 9.2	66.2 ± 9.5	0.199	65.0 ± 8.6	65.8 ± 9.7	66.0 ± 10.1	0.714	62.8 ± 9.7	64.8 ± 8.3	66.6 ± 8.1	0.088
Day-night HTN,%	59(45.4)^*^	101(53.4)^*^	122(67.8)^^+^	<0.001	46(51.7)^*^	67(58.3)	86(70.5)^^^	0.016	13(31.7)^*^	34(45.9)	36(62.1)^^^	0.011
INH, %	41(31.5)	48(25.4)	39(21.7)	0.145	32(36.0)^*^	33(28.7)	25(20.5)^^^	0.044	9(22.0)	15(20.3)	14(24.1)	0.868

ABPM: Ambulatory Blood Pressure Monitoring; Se Obese: Severely Obese; INH: Isolated Nocturnal Hypertension

*: *P* < 0.05 vs. Severe obesity, +: vs. obesity ^ : vs. overweight

**Fig. 3:**
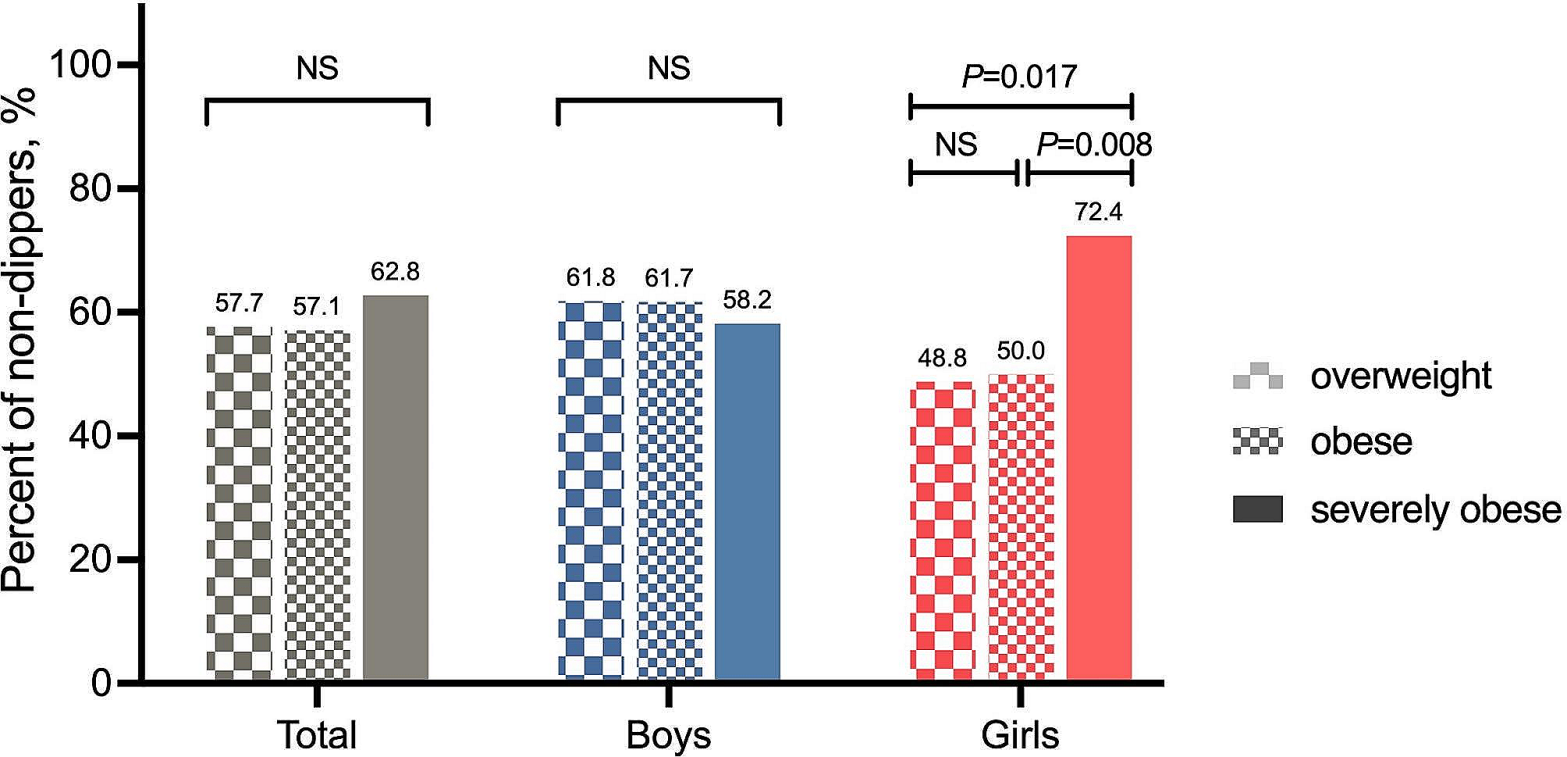
Prevalence of non-dippers in different degrees of obesity grouped by sex. Columns from light to dark represented the increasing severity of obesity. Statistical significance was assessed using the Chi-squared test and Bonferroni correction was used in multiple comparisons

Figure 3 shows the prevalence of non-BP dipping in participants at each stage of obesity. Among the 499 participants, the prevalence of non-dippers did not significantly differ among obesity categories and this phenomenon also occurred in boys. However, the dipping status of girls seemed to be associated with the severity of adiposity. Compared with their overweight and obese counterparts, girls who were severely obese demonstrated a higher prevalence of non-dipping status in BP (72.4% vs. 48.8%, *P* = 0.017; 72.4% vs. 50.0%, *P* = 0.008). This finding was further confirmed by multi-variable Logistic regression analysis (Table 3). After adjustment for age and OBP, the relationship between severely obese and non-dipping patterns remained significant in girls (*P* = 0.029).

**Table 3:** Multivariable logistic regression analysis for non-dipping pattern in different genders

	Non-dipping pattern (Boys)			Non-dipping pattern (Girls)		
	β	B(95% CI)	*P*	β	B(95% CI)	*P*
Age	0.047	1.048 (0.905–1.214)	0.531	0.082	1.085 (0.885–1.331)	0.431
Obesity Degree			0.879			0.023
Obese	0.029	1.029 (0.579–1.831)	0.922	-0.027	0.973 (0.447–2.118)	0.945
Severely Obese	-0.101	0.904 (0.509–1.603)	0.729	0.962	2.616 (1.105–6.191)	0.029
Elevated OBP	-0.211	0.810 (0.330–1.988)	0.531	1.428	4.171 (0.446–39.011)	0.211

OBP: Office Blood Pressure

## Discussion

In present study, we found that the relationship between severity of obesity and BP dipping status was sex-dependent. Compared with girls who were overweight and obese, non-dipping patterns were more frequent in girls with severely obesity, whereas this relationship was not identified in boys. In addition, an interaction effect between sex and the severity of obesity was found in nighttime SBP. As obesity stage increased, girls experienced a more evident rise in night-time BP than boys, which might account for the higher prevalence of non-dipping status in severely obese girls.

The mechanism connecting adiposity and non-dipping patterns in BP is still under active investigation. Accumulating evidence suggests that factors which contribute to elevated BP in obese individuals, such as insulin resistance, impaired renal-sodium handling, inappropriate sympathetic nervous system (SNS) activity and abnormal renin-angiotensin-aldosterone system (RAAS) activity could also lead to abnormal BP dipping patterns [26–29]. Besides, some of the common comorbidities in individuals with overweight or obesity may worsen the non-dipping status through mechanisms that are independent of obesity. For example, fragmented sleep caused by obstructive sleep apnea syndrome (OSAS) may directly increase BP level at night. Depression, a frequent psychological comorbidity of excessive adiposity, is associated with disrupted biological clock, therefore might interfere with BP circadian patterns as well [30–33]. In addition to directly and indirectly causing non-dipping status, overweight/obese status could enhance the unfavorable impact of certain conditions on nocturnal BP fall. A recent study found that the influence of OSAS on pediatric BP was dependent on weight and being overweight/obese could synergistically worsen nocturnal BP in children with OSAS [34].

Abnormal BP dipping status in adolescents could lead to TOD at an early age and increase the risk of coronary atherosclerosis in adulthood [8–10, 35]. As a major risk factor of non-dippers, determination of the severity of obesity might help physicians identify adolescents who are at greater risk for this condition. Several studies have investigated the relationship between the degree of adiposity and dipping status in young people, but their findings were mixed. In a study of 247 obese subjects from 5 to 21 years old, Macumber et al. failed to find a significant association between non-dipping patterns and the severity of obesity, which they assumed might have been due to inadequate statistical power [14]. Similar result was obtained in a Turkish cohort, which also consisted of a small sample of 63 adolescents [18]. Conversely, in a recent study of 263 young participants, there was a higher prevalence of non-dippers among individuals with more advanced obesity. In addition, elevated left ventricular mass, a biomarker closely associated with non-dipping patterns, was also identified in individuals with marked obesity [17]. Despite these conflicting results, these studies did not consider the possibility of sex-specific manifestations. In another research carried out in Sweden, Framme.et al found that compared with lean counterparts, obese adolescent girls experienced less nocturnal fall in BP while such difference was not detected in boys, suggesting that the association between dipping status and obesity might be sex-specific in adolescents [36]. However, this study had a small sample and only include 29 boys. On the contrary, there were nearly twice as many boys as girls in the present cohort, yet a significant association between SBP dipping and obesity was observed only in girls. Compared with their less obese counterparts, girls with severe obesity were more likely to develop non-dipping patterns.

We noted that our findings of sex differences in the relationship between nocturnal dipping status and obesity degrees could be quite surprising. At first sight, this finding seems to conflict with the widely accepted opinion that young women are at lower cardiovascular risk than age-matched men, due to the protection of estrogen [26]. Estrogen has been shown to have anti-proliferative effects on vascular smooth muscle cells thus could inhibit the development of hypertension [37]. However, this cardioprotective effect is limited by obesity, and therefore female could be more vulnerable in such cases [26]. A population study showed that compared with obese men, women who were premenopausal but obese, were at higher risk of developing hypertension [38]. In adolescents, a stronger relationship between adiposity and left ventricular abnormalities was also found in girls [39, 40]. This increased susceptibility in women with obesity could be partly explained by the differences in fat mass and distribution between genders. Compared with men of similar BMI, women have substantially higher adipose mass [41]. A recent study also found that adolescent girls had more neck fat than boys while neck adiposity has been proved to promote OSAS in adults [42].

Besides cardiovascular risk, sex differences observed in other obesity-related conditions might also be associated with our findings. According to a large-scale research in urban China, shorter sleep duration, a situation closely related with elevated nocturnal BP, increased the risk of adiposity in girls but not in boys [43]. Studies from Korea and Norway also found that compared with boys, girls troubled by weight problems were more likely to feel stress and develop irregular eating habits [20, 21]. These unhealthy emotions and behaviors might disturb their biological clocks, leading to abnormalities in BP circadian patterns [30, 32, 33, 44].

There are some limitations. First, although we were able to clearly define individuals as overweight, obese, or severely obese using their BMI, we did not have waist circumference data, which also reflected the degree of obesity. Second, besides abnormalities in BP, other complications of obesity, such as OSAS and diabetes, were not recorded and analyzed in the present study. Third, given the cross-sectional design, we failed to explore the longer-term effect of the gender differences identified in the present study. We hoped that longitudinal follow-up studies could be conducted in the future.

In conclusion, for overweight and obese adolescent girls, increasing severity of obesity is associated with a higher risk of non-dipping status in blood pressure. However, this relationship is not present in boys. These findings add to the accumulating evidence regarding sex-related differences in pediatric cardiometabolic disorders. For severely obese girls, performing ABPM routinely is needed and decreasing the severity of adiposity might reduce the risk of developing abnormal BP dipping patterns.

## Data Availability

The data underlying this article will be shared on reasonable request to the corresponding authors.

## Abbreviations

ABPM: Ambulatory blood pressure monitoring
TOD: Target organ damage
LVH: Left ventricular hypertrophy
SNS: Sympathetic nervous system
RAAS: Renin-angiotensin-aldosterone system
OSAS: Obstructive sleep apnea syndrome
